# Shear wave elastography imaging of carotid plaques: feasible, reproducible and of clinical potential

**DOI:** 10.1186/1476-7120-12-49

**Published:** 2014-12-08

**Authors:** Kumar V Ramnarine, James W Garrard, Baris Kanber, Sarah Nduwayo, Timothy C Hartshorne, Thompson G Robinson

**Affiliations:** Department of Medical Physics, University Hospitals of Leicester NHS Trust, Sandringham Building, Level 1, Leicester Royal Infirmary, Infirmary Square, Leicester, LE1 5WW UK; Department of Cardiovascular Sciences, University of Leicester, Leicester, UK; Department of Vascular and Endovascular Surgery, University Hospitals of Leicester NHS Trust, Leicester, UK; National Institute for Health Research (NIHR) Biomedical Research Unit (BRU) for Cardiovascular Sciences, University of Leicester, Leicester, UK

**Keywords:** Ultrasound, Carotid, Plaque, Stenosis, Shear wave, Elastography, Elastogram

## Abstract

**Background:**

Shear Wave Elastography (SWE) imaging is a novel ultrasound technique for quantifying tissue elasticity. Studies have demonstrated that SWE is able to differentiate between diseased and normal tissue in a wide range clinical applications. However its applicability to atherosclerotic carotid disease has not been established. The aim of this study was to assess the feasibility and potential clinical benefit of using SWE imaging for the assessment of carotid plaques.

**Methods:**

Eighty-one patients (mean age 76 years, 51 male) underwent greyscale and SWE imaging. Elasticity was quantified by measuring mean Young’s Modulus (YM) within the plaque and within the vessel wall. Echogenicity was assessed using the Gray-Weale classification scale and the greyscale median (GSM).

**Results:**

Fifty four plaques with stenosis greater than 30% were assessed. Reproducibility of YM measurements, quantified by the inter-frame coefficient of variation, was 22% within the vessel wall and 19% within the carotid plaque. Correlation with percentage stenosis was significant for plaque YM (p = 0.003), but insignificant for plaque GSM (p = 0.46). Plaques associated with focal neurological symptoms had significantly lower mean YM than plaques in asymptomatic patients (62 kPa vs 88 kPa; p = 0.01). Logistic regression and Receiver Operating Characteristic (ROC) analysis showed improvements in sensitivity and specificity when percentage stenosis was combined with the YM (area under ROC = 0.78).

**Conclusions:**

Our study showed SWE is able to quantify carotid plaque elasticity and provide additional information that may be of clinical benefit to help identify the unstable carotid plaque.

**Electronic supplementary material:**

The online version of this article (doi:10.1186/1476-7120-12-49) contains supplementary material, which is available to authorized users.

## Background

Carotid artery plaque is a major risk factor for stroke, a leading cause of death and of adult disability worldwide. Although landmark European and North American clinical trials have shown carotid endarterectomy to be beneficial for symptomatic patients with stenosis over 70% [1], the benefits of carotid endarterectomy for moderate stenosis patients and for asymptomatic patients continues to be debated. For a severe stenosis (≥70%), the number needed to treat (i.e. the number of operations required to save one life at 5 years) has been estimated as 6, which increases for moderate stenosis (50-69%) to 15 [2]. As carotid endarterectomy has a 5% risk of an intra-operative stroke, there is considerable interest in developing improved methods of identifying the unstable plaque in order to improve clinical risk stratification for predicting stroke.

Shear Wave Elastography (SWE) is a novel imaging technique developed to assess tissue elasticity and to differentiate diseased tissue from normal tissue. Shear Wave Elastography exploits acoustic radiation force to generate shear wave propagation in tissue [3]. Application of a theoretical model of wave propagation enables the quantification of the Young’s Modulus (YM) from the measurement of shear wave velocity. Studies have shown potential clinical applications in a wide range of tissues including the breast, thyroid, liver, prostate, spleen, salivary glands, and cervical lymph nodes [4] but there are very few vascular studies [5, 6]. Application of SWE for imaging carotid plaques is particularly challenging as the carotid plaque is often small, of variable morphology, subject to arterial pulsatile motion, with heterogeneous surrounding tissue. Despite these challenges, we have previously shown that SWE estimates of YM using in-vitro vessel stenosis phantom models are feasible, and able to distinguish soft and hard regions with good reproducibility [7]. Also, our case study demonstrated the clinical application of SWE for imaging carotid plaques in comparison with the greyscale median (GSM) and histological assessment [6].

The purpose of this clinical study was to investigate the feasibility of using SWE imaging for measuring the YM of carotid plaques and the potential clinical value of SWE imaging to help identify the unstable carotid plaque. The underlying hypothesis behind this study is that unstable carotid plaques can be identified by characterisation of the carotid plaque stiffness.

## Methods

The study was approved by the National Research Ethics Service (NRES) Committee East Midlands - Northampton (reference 11/EM/0249), followed institutional guidelines, and each patient gave informed consent before participating in the study. Eighty-one patients undergoing a clinical carotid ultrasound scan were recruited from the rapid-access TIA Clinic and the Vascular Studies Unit. Patients were defined as atherosclerotic if they had carotid artery stenosis of ≥30% (North American Symptomatic Carotid Endarterectomy Trial (NASCET) criteria) measured using blood flow velocities in addition to B-mode diameter measurements and color Doppler findings. Plaques were classified as either having caused focal neurological symptoms relating to the ipsilateral brain hemisphere within the past six-month period (i.e. symptomatic) or as asymptomatic following specialist review of patient symptoms and clinical or radiological (computed tomography and/or magnetic resonance imaging) findings by the stroke physician. This classification based on symptoms was used as a surrogate marker of plaque vulnerability in this study.

### Data collection

All patients underwent routine clinical B-Mode ultrasound imaging with a Philips IU-22 scanner and L9-3 probe (Philips Healthcare, Eindhoven, The Netherlands), to assess the degree of stenosis using the NASCET criteria [1]. A 10-second cine-loop of the longitudinal section of the carotid plaque was acquired. SWE ultrasound imaging was performed using an Aixplorer® scanner with a L15-4 MHz linear array probe (Supersonic Imagine, Aix en Provence, France) and 10-seconds of cine-loop data were acquired. The scanner SWE settings were standardised to optimise acquisitions based on initial clinical experience as follows: acoustic power (maximum); smoothing (mid-range setting of 6); persistence (off); gain (65-70%); SWE option (penetration). The SWE region of interest box size was adjusted to include the carotid plaque/stenosis (typically 1 cm width by 2 cm length). Blue and red areas on the elastogram corresponded to low YM (soft) and high YM (stiff), respectively, up to a maximum stiffness of 300kPa. In the case of patients without atherosclerosis, the images were acquired at the carotid bifurcation. Detailed description of the adopted SWE technique is provided elsewhere [8]. Briefly, visualization of soft tissue viscoelastic properties was achieved by using ultrasonic focused beams to remotely generate mechanical vibration sources radiating low-frequency, shear waves inside tissues. The Aixplorer® scanner both generated and estimated the propagation speed of the resulting shear waves in order to calculate and display the YM, based on the formula YM = ρc^2^, where YM is tissue elasticity, ρ is tissue density and c is shear-wave velocity.

### Image analysis

**Figure 1:**
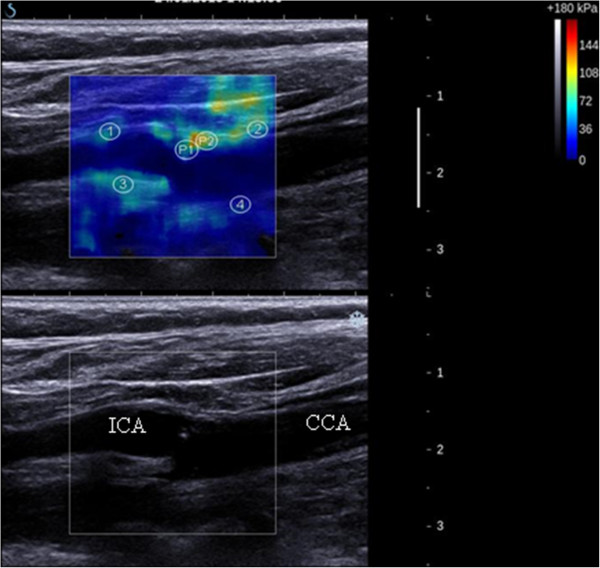
**Image of a grade 1, 30-40% stenosis at the origin of the internal carotid artery (above: elastogram; below: B-mode image).** Examples of selected ROIs are shown for this patient with atherosclerosis. The 4 ROIs from within the wall are labelled [1–4] as are the 2 from within the plaque (P1 and P2). The internal carotid artery (ICA) and the common carotid artery (CCA) are labelled.

The SWE frame rate of 1Hz provided approximately 10 frames of SWE data per acquisition. The first two SWE frames were discarded in order to allow the SWE acquisition to settle and reduce variability and, and five remaining frames were selected for analysis. In-built analysis software (Q-box tool) on the Aixplorer® scanner was used to quantify the mean YM within and across multiple circular regions-of-interest (ROIs) of 2 mm radius each, in order to sample tissue stiffness over the plaque body, as seen in Figure 1. Greyscale median (GSM), a quantitative measure of ultrasonic echogenicity of plaques, were calculated and normalised using previously described methods [9]. Briefly, our in-house MATLAB program (MathWorks, Natick, Massachusetts, USA) combined speckle-tracking techniques and a novel surface detection algorithm, based on a probabilistic approach to lumen segmentation [10], to semi-automatically assess dynamic variations in plaque characteristics, and track the plaque outline to estimate mean GSM across all frames. The scan was optimised by the operator, however the time-gain compensation (TGC) was standardised to remain linear for all depths corresponding to the carotid artery. Standard B-Mode acquisition settings were: Vasc Car™ preset, persistence low, XRES™ and SONOCT™ on, with a mean frame rate of 32 Hz. Subjective assessment of plaque appearance was performed by two independent observers, in accordance with the Gray-Weale classification. This classification graded plaques on a scale from 1 to 4 based on the echogenicity of the plaque from the B-Mode image [11]. A fifth grade added by Geroulakos et al. [12] was used for heavily calcified plaques that could not be graded.

### Statistical analysis

All statistical analyses were carried out using GRAPHPAD PRISM® version 6 (Prism, California, USA) and SPSS software version 20 (IBM Corporation, New York, USA). Demographic analysis was performed to highlight risk factors significantly associated with the presence of atherosclerosis in all patients and with the occurrence of symptoms in patients with coronary artery disease (CAD). Linear regression and Pearson correlation coefficient (PCC) were used to quantify any relationships between existing ultrasound methods and the novel SWE technique used in this study. Patient demographics and risk factors were assessed using Fisher tests for nominal data and student t-tests for the continuous data which was shown to be parametric. Logistic regression combined with receiver operator characteristics (ROC) curves and the area under the curve (AUC) was used to assess the clinical diagnostic potential. The reproducibility of estimates was assessed by calculating the mean and 95% confidence intervals (CI) and the mean inter-frame coefficient of variation (CV).

## Results

Forty-seven patients had atherosclerosis and 54 plaques of ≥30% stenosis were analysed, due to the occurrence of bilateral stenosis in seven patients. Six patients had unilateral internal carotid artery (ICA) occlusion, and were excluded from this study. Successful analysis was carried out on 52/54 (96%) of plaques. Data collection was unsuccessful in two plaques, both in obese patients, owing to attenuation of the ultrasound by superficial tissue.

Table 1 summaries patient demographics, grouped into patients with no or minor atherosclerosis (0-30%) stenosis, and patients with >30% atherosclerosis. Table 1 shows age, a history of smoking and a positive vascular diagnosis (transient monocular blindness, transient ischaemic attack and/or stroke) were associated with the presence of (>30%) atherosclerosis (p < 0.05). Only smoking remained significant when comparing the occurrence of symptoms in those patients with atherosclerosis (>30%). Other risk factors including diabetes mellitus, hypertension, hypercholesterolaemia and atrial fibrillation (AF) were not significantly different between groups. The demographics for symptomatic and asymptomatic patients are summarised in Table 2.

**Table 1:** **Summary of patient demographics** ^**1**^

Risk factor		Atherosclerosis (>30%)	No atherosclerosis/atherosclerosis (<30%)	p- value
**Number (n)**		47	34	-
**Gender (male: female)**		29:18	22:12	-
**Age**		76 ± 11 [52–99]	67 ± 12 [30–86]	0.002*
**BMI (kg/m** ^**2**^ **)**		27.0 ± 4.1 [20.9–35.7]	27.7 ± 4.3 [21.1–40.8]	0.61
**Diagnosis (n)**	**Vascular**	34 [72%]	16 [47%]	0.0363*
	**Non- vascular**	13 [28%]	18 [53%]	
**Smoking (n)**	**Current**	21 [44%]	9 [26%]	0.0006*
	**Ex- smoker**	13 [28%]	2 [6%]	
	**Never**	13 [28%]	23 [68%]	
**ABCD** ^**2**^ **score**		3 ± 2 [1–7]	3 ± 2 [0–6]	0.48
**Hypertension (n)**		31 [66%]	18 [53%]	0.64
**Hypercholesterolaemia (n)**		22 [47%]	16 [47%]	1.00
**Diabetes mellitus (n)**		13 [28%]	9 [26%]	0.45
**IHD/ PVD (n)**		11 [23%]	5 [15%]	0.41
**Family history (n)**		13 [28%]	7 [21%]	0.29
**Atrial fibrillation (n)**		7 [15%]	2 [6%]	0.60
**Previous TIA/stroke (n)**		21 [45%]	8 [24%]	0.06

^1^Values are given as the mean ± SD, [range]. BMI: Body Mass Index; IHD: Ischaemic Heart Disease; PVD: Peripheral Vascular Disease; TIA: Transient Monocular Blindness. The percentage of patients is also given. Statistically significant p-value (<0.05) is indicated by *.

**Table 2:** **Summary of patient demographics between symptomatic and asymptomatic patients** ^**1**^

Risk factor		Symptomatic	Asymptomatic	p- value
**Number (n)**		27	20	-
**Gender (male: female)**		18:9	11:9	-
**Age**		74 ± 11 [52–95]	77 ± 11 [60–99]	0.23
**BMI (kg/m** ^**2**^ **)**		28.0 ± 4.3 [23.1–35.7]	25.8 ± 4.1 [20.9–31.7]	0.33
**Smoking (n)**	**Current**	15 [56%]	6 [30%]	0.04*
	**Ex- smoker**	8 [29%]	5 [25%]	
	**Never**	4 [15%]	9 [45%]	
**ABCD** ^**2**^ **score**		3 ± 2 [1–5]	3 ± 2 [1–7]	0.89
**Hypertension (n)**		17 [63%]	14 [70%]	0.76
**Hypercholesterolaemia (n)**		14 [52%]	8 [40%]	0.56
**Diabetes mellitus (n)**		8 [29%]	5 [25%]	1.00
**IHD/PVD (n)**		6 [22%]	5 [15%]	1.00
**Family history (n)**		10 [37%]	3 [21%]	1.00
**Atrial fibrillation (n)**		4 [15%]	3 [15%]	1.00
**Previous TIA/stroke (n)**		13 [48%]	9 [45%]	1.00

^1^Values are given as the mean ± SD, [range]. BMI: Body Mass Index; IHD: Ischaemic Heart Disease; PVD: Peripheral Vascular Disease. The number and percentage of patients is also given. Statistically significant p-value (<0.05) is indicated by *.

**Figure 2:**
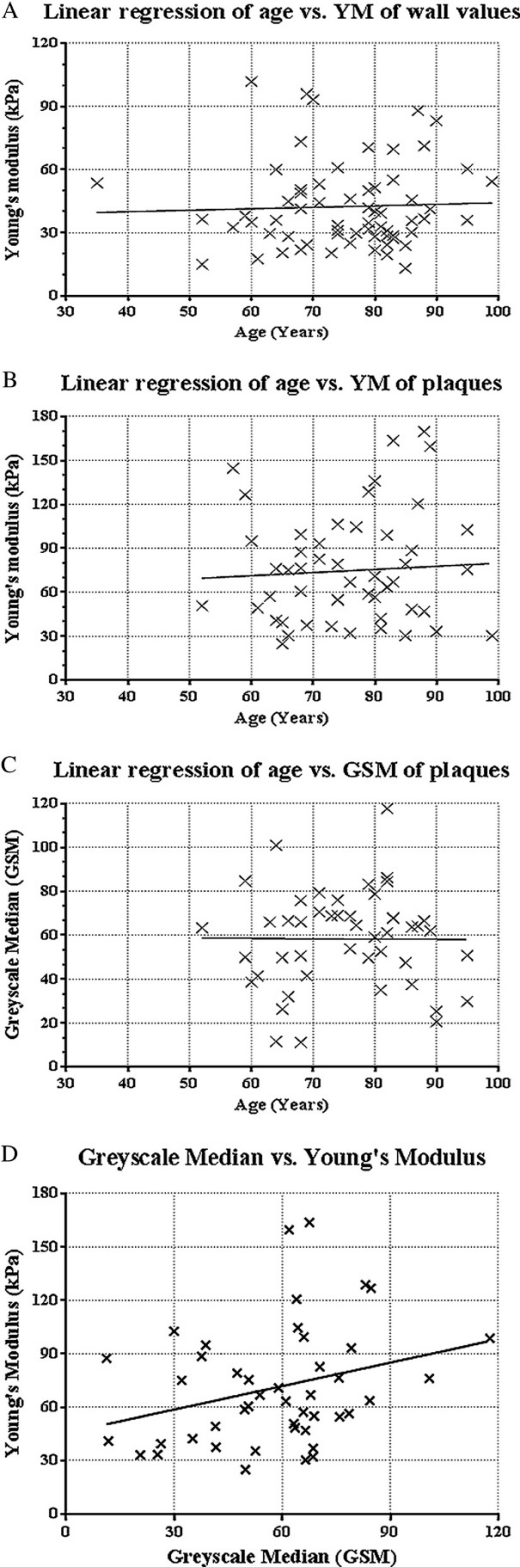
**Scatter plots, with line of best fit. A)** Shows age against the mean Young’s Modulus of arterial wall. **B)** Shows the age against the Young’s Modulus of all plaques. **C)** Shows age against the Greyscale median (GSM) of plaques. **D)** Shows the GSM of plaques against the YM of plaques.

Figure 2 shows scatter plots of age compared to YM in vessel wall and carotid plaque, and GSM of plaque. There was no significant relationship between age and YM with respect to the vessel wall (r^2^ = 0.002; p = 0.91) or YM within plaques (r^2^ = 0.004; p = 0.66), and no significant relationship between age and GSM of plaques (r^2^ = 0.00005; p = 0.96).

### Plaque characteristics

**Figure 3:**
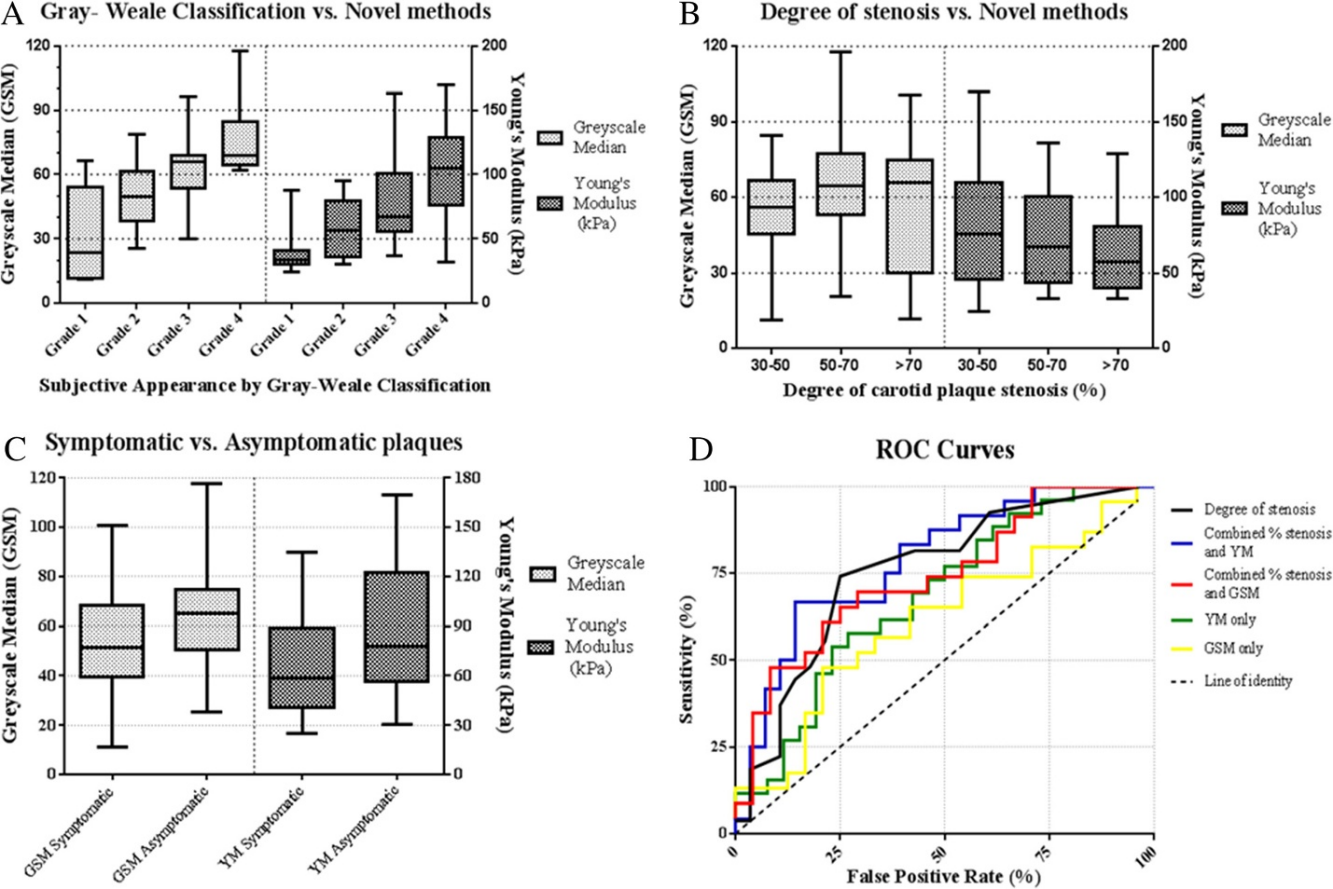
**Graphs illustrating key results. A)** Box and whisker plots showing the Greyscale median (GSM) and Young’s Modulus (YM) of plaques against subjective Gray-Weale Classification. Both values increase with higher classification of plaque appearance. **B)** Box and whisker plots illustrating plaque GSM and YM against the percentage stenosis, grouped into either mild (30-50%), moderate (50-70%) or severe (>70%). **C)** Box and whisker plots illustrating the plaque GSM and YM of symptomatic and asymptomatic plaques. **D)** ROC curves for the logistical regression of different ultrasound methods, and percentage stenosis as an individual method.

Plaques from symptomatic patients were generally less echogenic and the degree of stenosis was greater than plaques from asymptomatic patients (Table 3). There was a significant relationship between the Gray-Weale plaque classification and YM, (p < 0.001), and plaque classification and GSM (p < 0.001), Figure 3A. There was also a significant correlation between percentage stenosis and lower plaque YM (p = 0.003) but not between plaque GSM and percentage stenosis (p = 0.46), Figure 3B. The mean plaque YM of the symptomatic patients (mean 62 kPa; 95% CI = 51 – 73 kPa) was significantly lower (p = 0.01) than the mean plaque YM of the asymptomatic patients (mean 88 kPa; 95% CI = 71 – 105 kPa), Figure 3C. The mean plaque GSM in symptomatic patients (mean 54; 95% CI = 44 – 64) was not significantly different (p = 0.19) than the mean plaque GSM in asymptomatic patients (mean 62; 95% CI = 54 – 71), Figure 3C. There was no significant difference in the variance of results between the two groups (p = 0.65) and both plaque YM and GSM were normally distributed. Figure 2D shows a scatter plot comparing the GSM to the YM of all plaques. A weak relationship was found between increasing YM and GSM (r = 0.29), which was of borderline significance (p = 0.051).

Figure 3D shows the ROC curves plotted for the percentage stenosis, YM and GSM of plaques. The percentage stenosis was the best individual predictor of occurrence of symptoms, with the highest individual area under the curve (AUC = 0.75), followed by YM (AUC = 0.69) and GSM (AUC = 0.61). Combining percentage stenosis and YM improved the AUC value to 0.78. However when percentage stenosis was combined with GSM, the ROC curve gave a lower AUC value of 0.74 indicating no diagnostic improvement compared to percentage stenosis only.

**Table 3:** **The number of plaques by Gray-Weale classification** [9, 10] **and by stenosis grading**

Classification/grading		Symptomatic patients	Asymptomatic patients
**Gray-Weale**	1	5	2
	2	8	6
	3	10	11
	4	4	8
	5	0	0
**Percentage stenosis**	30-49	7	20
	50-69	10	4
	>70	10	3*

*Not including the 6 occluded carotid arteries.

### Reproducibility

Table 4 provides a summary of YM and GSM values in vessel wall and plaque, and the reproducibility of measurements across all patients, irrespective of clinical history or clinical examination. The mean YM within the wall was 33 kPa lower than in the plaque. The reproducibility of YM estimates as quantified by the mean inter-frame CV was 22% within the wall and 19% within the plaque.

**Table 4:** **Summary of YM and GSM values in vessel wall and plaque and inter-frame reproducibility of measurements** ^**1**^

All patients	Wall YM	Plaque YM	Plaque GSM
**Mean**	42 kPa (95% CI: 37-48 kPa)	75 kPa (95% CI: 64-85 kPa)	56 (95% CI: 52–65)
**CV**	22% (95% CI: 20-24%)	19% (95% CI: 17-21%)	7% (95% CI: 5-8%)

^1^The mean and 95% confidence intervals (CI) of results across all patients are shown in addition to the average inter-frame coefficient of variation (CV) of YM and GSM measurements.

Two additional supplementary video files are provided as examples of SWE acquisitions. Additional file 1 shows an ultrasound B-mode and SWE video clip of carotid plaque causing a ≥70% stenosis. Greyscale imaging demonstrates apparently large fibrous and calcified plaque corresponding to relatively high YM in the SWE image. Additional file 2 shows a video clip of a minor carotid plaque. Greyscale imaging demonstrates a predominately anechoic type 1 plaque on the posterior wall corresponding to relatively low YM in the SWE image.

## Discussion

This was the first study to demonstrate the feasibility of SWE imaging for the assessment of tissue elasticity in a range of carotid plaque stenoses and vessel walls. Our findings have demonstrated the potential clinical value of SWE imaging to help identify the unstable carotid plaque by using symptomatic patients as a surrogate measure of the unstable plaque in a retrospective study design.

SWE imaging of carotid plaques was successful in 52 out of 54 plaques giving an excellent technical sucess rate of 96%. The inter-frame reproducibility of YM estimates, represented by the inter-frame CV values was 22% for the vessel wall and 19% for carotid plaques. In comparison, our previous in-vitro phantom study produced CV values ranging from 8-20% [7]. Estimates of YM are affected by the pulsatile motion of the carotid arteries and the restricted temporal resolution of the commercial Aixplorer® scanner (1 Hz). Other clinical studies have quoted CV values of 12-17% in SWE assessment of liver [13] a clinical application, like breast, demonstrating growing clinical potential and good reproducibility [4]. SWE is considered less operator dependent, and with better reproducibility than earlier ultrasound elastography techniques that are based on operator compression of the tissue to induce a transient stress and assess tissue deformation [14]. Whilst the inter-frame reproducibility of YM may be clinically acceptable, the accuracy of YM estimates is dependent on the assumed relationship between shear wave velocity and YM. As discussed in previous studies, shear wave propagation in thin vessel walls and plaque is complex, and therefore the use of a different theoretical model than the commercial implementation adopted in this study is required for an accurate quantification of YM [7, 15]. Nevertheless, our study showing a mean YM across all plaques of 75 kPa (95% CI: 64-85 kPa) is comparable to previous measurements, although these are scarce. A recent study, directly measuring mechanical properties of carotid plaque tissue showed highly non-linear stretch–stress behaviour, with an increased stiffness with stretching due to the inherent microstructure. The YM of fibrous tissue had a large range from 6 to 891 kPa (median 30 kPa) and lipid ranged from 9 to 143 kPa (median 16 kPa) [16].

In our study, the YM of plaques correlated with plaque grading based on echogenicity using the Gray-Weale classification and this relationship might be expected, since previous work suggested that poorly reflective plaques contain more lipids [11]. Moreover, a significant relationship was evident between the YM of plaques and percentage stenosis. This relationship, however, may be the result of a confounding factor as the degree of stenosis of symptomatic plaques was generally higher than for asymptomatic plaques, and the mean YM of symptomatic plaques was significantly lower than asymptomatic plaque YM. Plaque GSM also correlated with the Gray-Weale classification. This association, which was higher than that observed with SWE, was expected as both methods are grades of plaque echogenicity. By contrast, and unlike YM, the GSM was not related to degree of stenosis. Although symptomatic plaques had lower GSM than asymptomatic plaques, this did not reach statistical significance (p = 0.19). This differs from previous studies, including our own, which find a significant association between lower GSM and symptoms [9, 17–19], and benefitted from larger sample size. Other studies [20] have also found no relation between the GSM and symptoms. Hence the value of GSM in evaluating plaque vulnerability continues to be debated and additional B-mode risk indices investigated [21].

The assessment of clinical potential for the ultrasound techniques used in this study suggested that percentage stenosis was the strongest individual marker for predicting symptomatic patients (AUC = 0.75). SWE estimates of YM were a better marker than GSM, and combining the YM with percentage stenosis yielded improved diagnostic performance (AUC = 0.78). Therefore, whilst quantifying plaque YM will not replace grading of plaque stenosis to determine eligibility for surgery, the additional information provided by SWE imaging in the vascular examination may help improve individual risk stratification. To benefit from the added value of SWE imaging of carotid plaque is straightforward, as implementation in the vascular ultrasound clinic is a simple extension to the current ultrasound examination.

Further work is required to substantiate our initial findings and to extend the study to assess additional parameters such as YM heterogeneity within plaque, a parameter which may further help identify the unstable carotid plaque. Advances in SWE technology, such as improved frame rate should improve reproducibility and enable assessment of temporal variation in YM through the cardiac cycle. Our retrospective study design used clinical symptoms as a surrogate measure of the unstable plaque. A large prospective multicentre study design is required to assess the diagnostic performance and prognostic ability of SWE imaging in identifying the unstable plaque and risk of stroke. This would provide the evidence required for implementation of SWE imaging technology into the routine vascular ultrasound clinic.

## Conclusions

Our study showed SWE is able to quantify carotid plaque elasticity and provide additional information that may be of clinical benefit to help identify the unstable carotid plaque.

## Electronic supplementary material

Additional file 1: **Ultrasound B-mode and SWE video clip of carotid plaque causing a ≥70% stenosis demonstrated at the origin to the ICA.** Greyscale imaging demonstrates apparently large fibrous and calcified plaque corresponding to relatively high YM in the SWE image. (ZIP 14 MB)

Additional file 2: **Ultrasound B-mode and SWE video clip of minor carotid plaque demonstrated at the carotid bifurcation.** Greyscale imaging demonstrates a predominately anechoic type 1 plaque on the posterior wall corresponding to relatively low YM in the SWE image. (ZIP 14 MB)

## Abbreviations

AF: Atrial fibrillation
AUC: Area under the curve
BMI: Body Mass Index
CAD: Coronary artery disease
CCA: Common carotid artery
CI: Confidence interval
CV: Coefficient of variation
GSM: Greyscale median
ICA: Internal carotid artery
IHD: Ischaemic Heart Disease
NASCET: North American Symptomatic Carotid Endarterectomy Trial
PCC: Pearson correlation coefficient
PVD: Peripheral Vascular Disease
ROC: Receiver operator characteristic
ROI: Region-of-interest
SWE: Shear Wave Elastography
YM: Young’s Modulus.
